# Passenger for millenniums: association between stenothermic microcrustacean and suctorian epibiont - the case of *Eurytemora lacustris* and *Tokophyra* sp

**DOI:** 10.1038/s41598-020-66730-2

**Published:** 2020-06-12

**Authors:** Łukasz Sługocki, Maciej Karpowicz, Agnieszka Kaczmarczyk-Ziemba, Joanna Kozłowska, Ingvar Spikkeland, Jens Petter Nilssen

**Affiliations:** 1 Department of Hydrobiology, Institute of Biology, University of Szczecin, Felczaka 3C, 71-712 Szczecin, Poland; 2 Center of Molecular Biology and Biotechnology, University of Szczecin, Wąska 13, 71-415 Szczecin, Poland; 3 Department of Hydrobiology, Faculty of Biology, University of Białystok, Ciołkowskiego 1J, 15-245 Białystok, Poland; 4 Department of Genetics and Biosystematics, Faculty of Biology, University of Gdańsk, Wita Stwosza 59, 80‐308 Gdańsk, Poland; 5 Østfold Museum Foundation, Dep. Haldenvassdragets Kanalmuseum, P.O. Box 64, NO-1870 Ørje, Norway; 6 Müller-Sars Society for Free Basic Research, Division of Ecology and Biogeography, P.O. Box 170, NO-4952 Risør, Norway

**Keywords:** Ecology, Zoology, Limnology

## Abstract

Epibionts often colonize the exoskeleton of crustaceans, which sometimes results in the development of a long-term relationship between them. Our present work confirmed that a specific epibiont is closely associated with the pelagic calanoid copepod *Eurytemora lacustris*, regardless of the region, which suggests a preserved interaction between these species. Molecular analyses revealed that the epibiont belongs to the genus *Tokophrya*. We also found that the level of basibiont colonization is related to its size and identified that the most intensely inhabited body parts are those located near the center of the copepod body. We hypothesize that the relationship between *Eurytemora* (basibiont) and *Tokophrya* (epibiont) was established during the Quaternary period, following which these two populations were fragmented into lakes where they survived in close interaction. In addition, we suppose that the close relationship between the two species indicates the coevolution of stenotherms. Further studies on the interactions between *Eurytemora lacustris* and *Tokophrya* are required in order to gain insight into the long-term relationship between the copepods and the epibionts.

## Introduction

Protozoan epibionts have often been reported to colonize the exoskeleton of crustaceans^1,2^. In addition, epibionts can affect the host communities by modulating the interaction between a host and the environment^3^. The colonization of a basibiont by an epibiont also leads to a significant change in the body surface of the former. Epibionts can settle on different parts of the host’s body, and their intensity of infection and prevalence most often depend on the availability of the basibionts^3^. It has been reported that epibionts benefit at the expense of their host and sometimes infect almost every specimen in a certain population^4^. Thus, epibionts exert multiple effects on copepods, which include a significant change in their sinking rates^1^, increase in their visibility to predators^5^, decrease in their swimming activity as well as mating success and survival rates^6^, and change in the symmetry of their body. It was shown that copepods that were inhabited by epibionts lived shorter under the conditions of food deficits, due to increased energy expenditure on movement^7^. Moreover, it has been experimentally proven that *Acartia hudsonica* nauplii that were inhabited by epibionts had lower rates of survival compared to uninfected nauplii^1^. In addition, significantly slower sinking rates were observed in infected individuals compared to uninfected ones. However, epibionts can also serve to protect the host specimens. A study demonstrated that a mixture of epibiont and basibiont chemical signals produced a repellent^3^. Moreover, the colonization of crustaceans by predatory suctorians may protect the host against those pathogens that in turn could serve as a food base for the epibionts. It seems that if the impact of epibionts on the copepods is adverse, then evolution would lead to the persistence of only uninfected copepod populations. These suggest that epibiosis is a multidimensional interaction between basibionts and epibionts, which is difficult to define and requires a better understanding.

Most of the studies on the epibiontic relationship between protozoa and microcrustacea available in the literature focus on peritriche ciliate^2,8,9^, while suctorians have been less often investigated^10,11^. Filter-feeding epibiontic peritriche are found at all latitudes and various habitats, and predator suctorians that live in interaction with microcrustaceans are usually detected in deep lakes, estuaries, and oceans^2,9^. Some features of crustaceans, such as color, texture, smell, or taste, can serve as a signal to an epibiont to identify its host location^3^. The apparent diversity of the microcrustaceans allows the epibionts to interact with the species appropriate for them, sometimes resulting in close interactions. Although the majority of epibionts are not host-specific, certain species have been observed to favor some microcrustaceans^9,12^. When a host organism encounters environmental stress associated with hypoxia or mechanical damage, or caused by the presence of pollutants or toxins, it becomes an inappropriate basibiont, and in such cases, the epibiont species must have a similar range of environmental tolerance as the host. As a result, a long-lasting specific interaction is established in some host–epibiont systems. Therefore, we investigated if the strong interaction of epibionts with basibiont can modulate the life histories of the host organisms.

**Figure 1:**
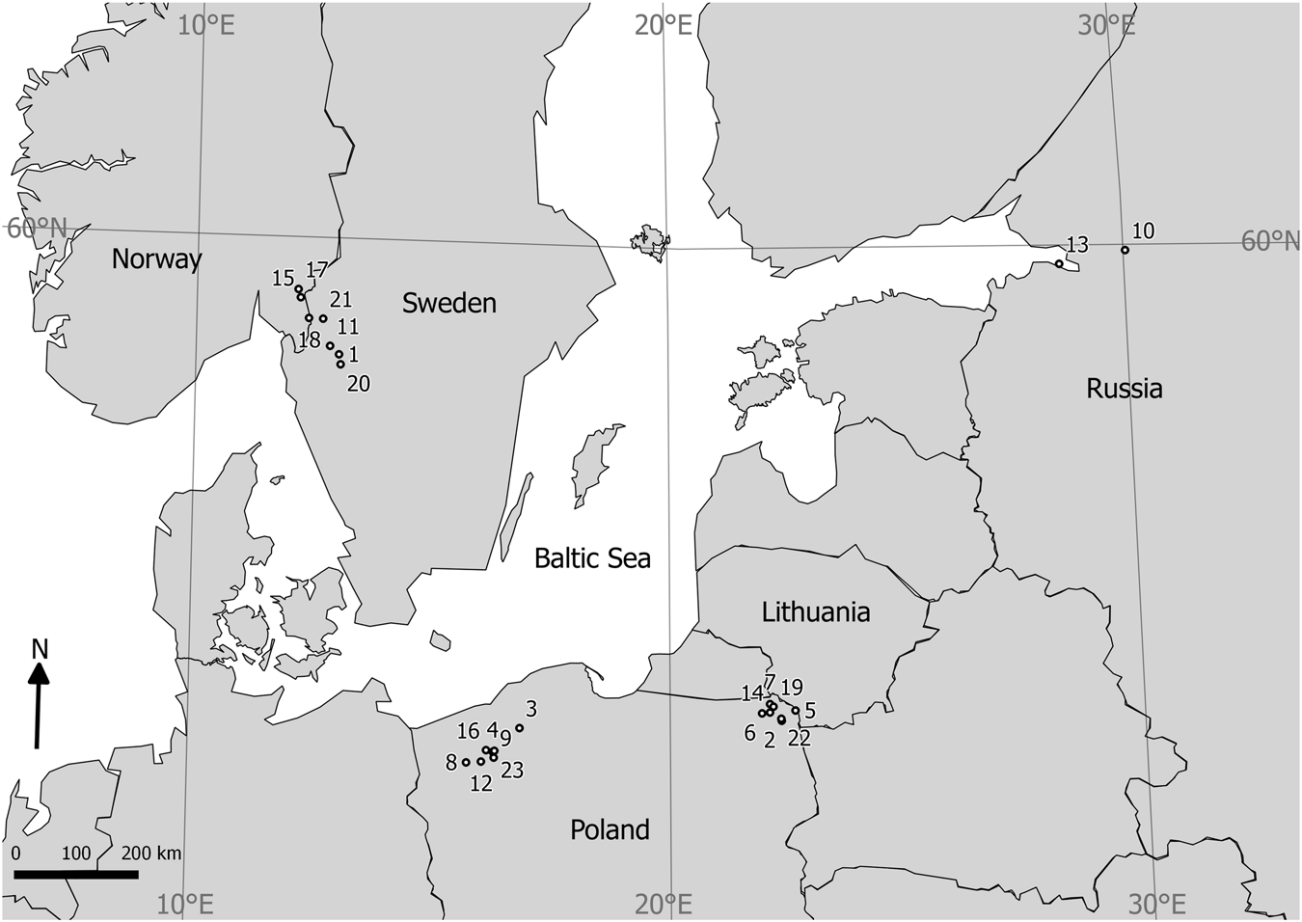
Map of the sampled water bodies. 1—Ånimmen; 2—Białe Wigierskie; 3—Cieszęcino; 4—Drawsko; 5—Gaładuś; 6—Garbaś; 7—Hańcza; 8—Ińsko; 9—Krzemno; 10—Ladoga; 11—Laxsjön; 12—Lubie; 13—Neva Bay; 14—Ożewo; 15—Rødenessjøen; 16—Siecino; 17—Skulerudsjøen; 18—Store Le; 19—Szurpiły; 20—Vänern; 21—Västra Silen; 22—Wigry; 23—Żerdno. A map was generated using QGIS version 2.18.24 (https://qgis.org/).

Among microcrustaceans, copepods are the most widespread and abundant, and offer a lot of space for the survival of epibionts. The original habitat of Copepoda was seawaters, but many taxa have evolved and adapted to freshwater habitats^13^. An example of the copepod genus that inhabits freshwater, brackish water, and saltwater is *Eurytemora*. The genetic and physiological mechanisms of freshwater invasions by the representatives of this genus are currently better understood^14,15^. During their transition from seawaters to freshwaters, certain populations of *Eurytemora* adapt to live in those habitats. At the end of the Quaternary period, changes in seawater levels caused by climate change resulted in the closure of isolated populations of *Eurytemora* in deep lakes. One example of such populations is *Eurytemora lacustris* (Poppe, 1887), a stenothermic copepod species that inhabits deep and clearwater lakes^16–19^ and is occasionally observed in estuarine waters connected with large freshwater lakes^20,21^. The latest report from NE Poland revealed that a specific suctorian ciliate inhabits on *E. lacustris* populations, while this epibionts are not found on other zooplankton species. Preliminary morphological reports indicated that the epibiont is similar to *Acineta tuberosa*^19^. Another similar epibiont was also found on *E. lacustris* isolated from the Ratzeburger Lake complex in Germany^17^ (Fig. 1B), indicating that this is not just a local phenomenon. Therefore, we decided to investigate more lakes in various geographical regions to confirm if a close relationship exists between these 2 species. We analyzed whether the interaction between the stenothermic copepod and the suctorian epibiont can result in a long-term relationship. In addition, we hypothesize that this epibiont could be a glacial relict that spread along with its hosts after the ice age. Therefore, we aimed to: (i) perform a genetic analysis of the epibiont and its phylogenetic affinity, (ii) compare its prevalence between different geographical regions, and (iii) determine the infection parameters of the basibiont and (iv) the patterns of body colonization.

## Methods

A total of 36 zooplankton samples were collected from 23 water bodies located in Central Europe (mainly Baltic Sea basin) during the years 2008–2019 (Fig. 1). Water bodies included in the study presents a range of maximum depth from 33 to 220 meters, therefore all are potentially the habitat of *Eurytemora lacustris*^17^. Only Neva Bay is the atypical habitat of *Eurytemora lacustris* because it is brackish waters. Due to the varied sampling efforts, the numbers of lakes, samples, and *E. lacustris* specimens that were investigated were not uniform and differed in each region (Scandinavia—7 lakes, 7 samples, and 131 specimens; NW Poland—7 lakes, 17 samples, and 1033 specimens; NE Poland—7 lakes, 8 samples, and 354 specimens; Russia—2 water bodies, 2 samples, and 90 specimens).

Zooplankton samples were collected from the hypolimnion of the lake to the surface using vertical hauls with plankton net (mesh of 100 µm; d = 20 cm). Concentrated samples were added into a 110-ml tube and fixed in 96% ethanol. The *Eurytemora* species were identified using a taxonomic key^22^. All water bodies were inhabited by only one species of *Eurytemora* with exception of Neva Bay where other congeners were present. *Eurytemora lacustris* populations that were taken for the present study were previously identified using *COI* and *cytb* sequences^21^. The specimens of *E. lacustris* (n = 1608) were selected in a plankton chamber using a Zeiss Primo Vert reverse microscope (Germany). Calanoids were transferred to glycerin and closed in slides. Copepods were identified and suctorians were counted in multiple digital images obtained using Nikon Eclipse 50i microscope (Japan) equipped with the software ToupView (ToupTek Photonics, China). The body size of the specimens was measured without taking the caudal setae into account. To determine the level of colonization of the suctorians on the surface of the copepods, the following body parts were distinguished: cephalosome (G), pedigerous somite (Th), genital somite and anal somite (Gs+As), and caudal ramus (Fu).

In some lakes, the number of specimens was not sufficient for calculating the prevalence. Therefore, we took into consideration the abovementioned 4 regions for assessing the geographic distribution of the suctorians (Scandinavia, NW Poland, NE Poland, Russia). Before performing analysis, we checked normal distribution using Shapiro-Wilk test. The simple relationship between the body size of *Eurytemora* and the number of epibionts was analyzed using Pearson’s correlation. To determine the region that was characterized by the highest rate of infection, we performed analysis of variance (ANOVA) type III sums of squares (SS) followed by Tukey’s honestly significant difference (HSD) test. The same ANOVA was also used for analyzing the differences in epibiont infection in terms of sexes and maturity stages. To determine most infected body parts of copepod by epibiont we performed Kruskal-Wallis test. To evaluate the differences in infection intensity in certain body parts of *Eurytemora*, with respect to sex, region, and body size of calanoids, a canonical correspondence analysis (CCA) was performed on the data collected from infected specimens.

Two separated populations of epibionts (Ińsko and Drawsko lakes) were considered for genetic analyses. Samples of infected basibionts were collected on the 20^th^ of September 2019. Ten cells were hand-picked from each sample and gathered for DNA extraction. The collected cells were transferred to sterile, distilled water, and then lysis buffer and proteinase K were added. DNA was isolated from the cells using Sherlock AX extraction kit (A&A Biotechnology, Poland) following the manufacturer’s protocol. The SSU rRNA gene was amplified using a Platinum™ II Hot-Start Green PCR Master Mix (Invitrogen, Thermo Fisher Scientific, USA) and primers^23^, under the following conditions: 2 min at 94 °C; 35 cycles of 15 sec at 94 °C, 15 sec at 58 °C, 30 sec at 68 °C; and final extension at 68 °C for 5 min. The amplification products were separated -by 1% agarose gel electrophoresis using a 1X SB buffer and visualized using SimplySafe (EURx, Poland) under UV light. All the PCR products were purified by treatment with alkaline phosphatase and exonuclease I (Thermo Fisher Scientific, USA) according to the manufacturer’s protocol and were then sequenced using the BigDye terminator cycle sequencing method.

All the newly obtained sequences were deposited in the GenBank database (accession numbers: MT276225-MT276228). For phylogenetic analyses, sequences of 11 SSU rRNA gene fragments from GenBank were used additionally, which belonged to the following species: *Acineta* sp., *Acineta compressa*, *A. tuberosa*, *Acineta flava*, *Tokophrya lemnarum*, *Tokophrya quadripartita*, *Tokophrya infusionum*, and *Tokophrya huangmeiensis* (Table 1). Two sequences belonging to the species *Loxodes magnus* and *Orthodonella apohamatus* (accession numbers: L31519 and DQ232761, respectively) were used as outgroups. The sequences were aligned using ClustalW algorithm implemented in MEGA7 software^24^. GBlocks was used for identifying and removing highly divergent regions as well as poorly aligned positions^25^. Phylogenetic analyses were conducted with the Maximum Likelihood approach using the MEGA7 software, by incorporating the GTR + I + G^26^ model of molecular evolution selected by JMODELTEST 2.1.10 as the best fit for the study data^27^. Bootstrap probabilities with 1000 pseudoreplicates were used for verifying the reliability of the clades in the phylogeny. The final phylogenetic tree was edited using FigTree software, version 1.4.2 (http://tree.bio.ed.ac.uk/software/figtree/).

**Table 1:** Accession numbers of the species used for the phylogenetic analyses.

Species	GenBank number	Species	GenBank number
*Acineta* sp.	AY332717	*Tokophrya lemnarum*	AY332720
*Acineta* sp.	AY332718	*Tokophrya lemnarum*	AY332720
*Acineta* sp.	AY332719	*Tokophrya quandripartita*	AY102174
*Acineta flava*	HM140400	*Tokophrya infusionum*	JQ723984
*Acineta compressa*	FJ865205	*Tokophrya huangmeiensis*	KJ567607
*Acineta tuberosa*	FJ865206		

## Results

### Infection distribution

We found that the suctorian epibionts of *E. lacustris* were widely distributed in the Baltic basin. Their mean prevalence in the assessed regions was as follows: 11% in Russia, 20% in NW Poland, and 31% in both NE Poland and Scandinavia. The suctorians were found throughout the research area, but their distribution varied between the lakes and ranged from 0 to 13% in Russia, 0 to 52% in Scandinavia, 0 to 62% in NW Poland, and 0 to 100% in NE Poland. The results of ANOVA type III SS revealed differences in the infection levels between regions (*F*_3,346_ = 41.6; *p* < 0.0001), while those of Tukey’s HSD test showed that infection level was the highest in NE Poland (*p* < 0.0001) compared to the other regions.

**Figure 2:**
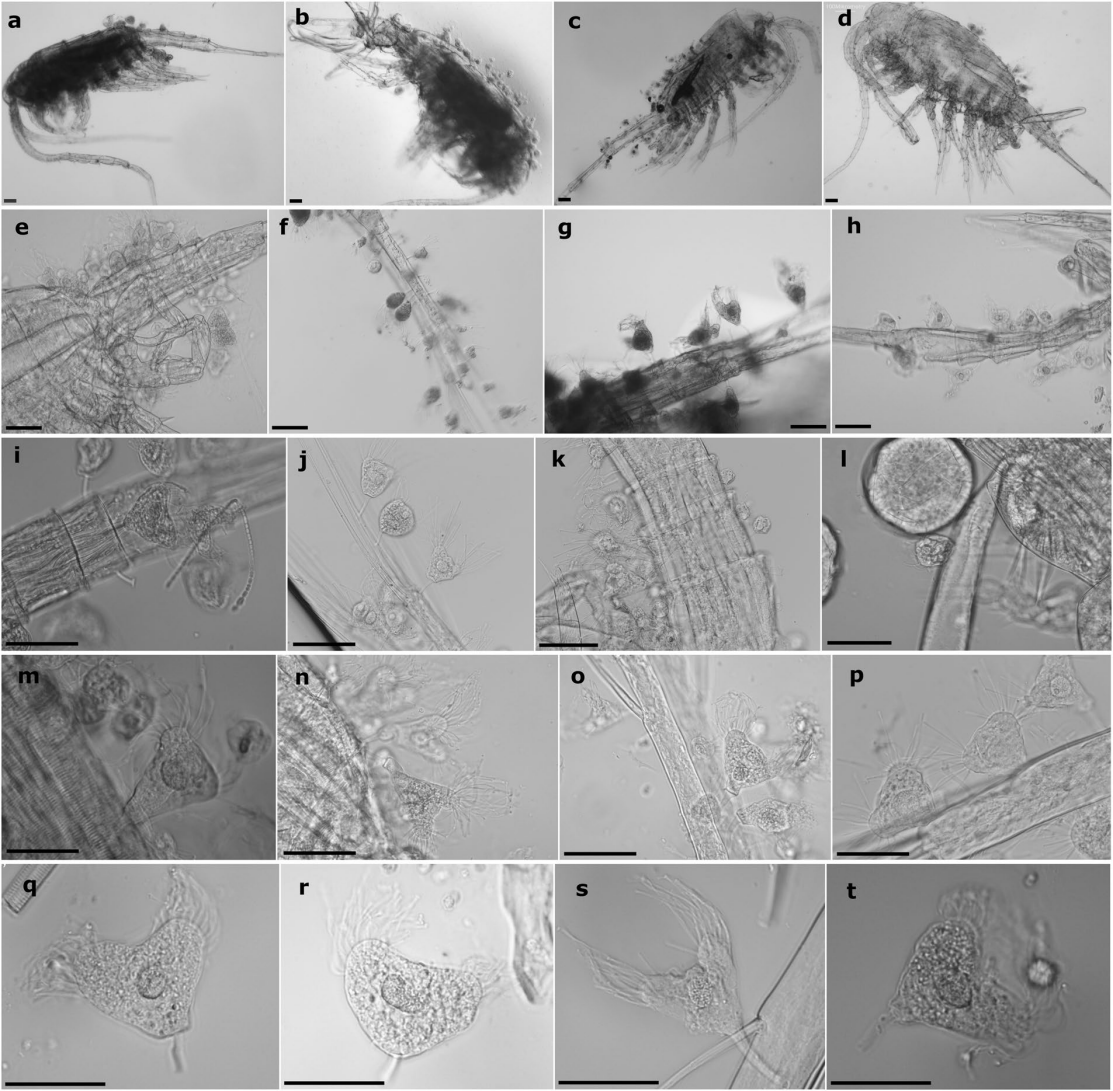
*Tokophrya* isolated from the assessed regions: NE Poland—F, J, K, and P; NW Poland—B, D, E, G, L, M, N, O, and S; Russia—C and T; Scandinavia—A, H, I, Q, and R. Scale bar: 50 µm.

In certain water bodies, we did not find any infected copepods, while in most of the studied lakes the number of *Eurytemora* was low (Ożewo—1 specimen, Ånimmen—8, Laxsjön—6, Skulerudsjøen—6, Rødenessjøen—4). In addition, no infected *Eurytemora* was found in the estuarine waters of Neva Bay (15 specimens). In Cieszęcino, a small and isolated lake, not a single infected *Eurytemora* was found, despite a large number of individuals (n = 200) were tested from 3 seasons (and observations recorded in previous years). Furthermore, the morphological assessment of the epibionts collected from the analyzed regions and lakes did not show any differences between the specimens (Fig. 2), and therefore, we assumed that it was the same species.

### Genetic affinities of epibiont

The newly obtained sequences of 18 S rRNA were compared with those deposited in the GenBank database. The search results showed their close match (94%) with the sequence of *T. quadripartita* (Claparède & Lachmann, 1859) Bütschli, 1889, whereas their more distant match (85%) to *A. flava* Kellicott, 1885.

**Figure 3:**
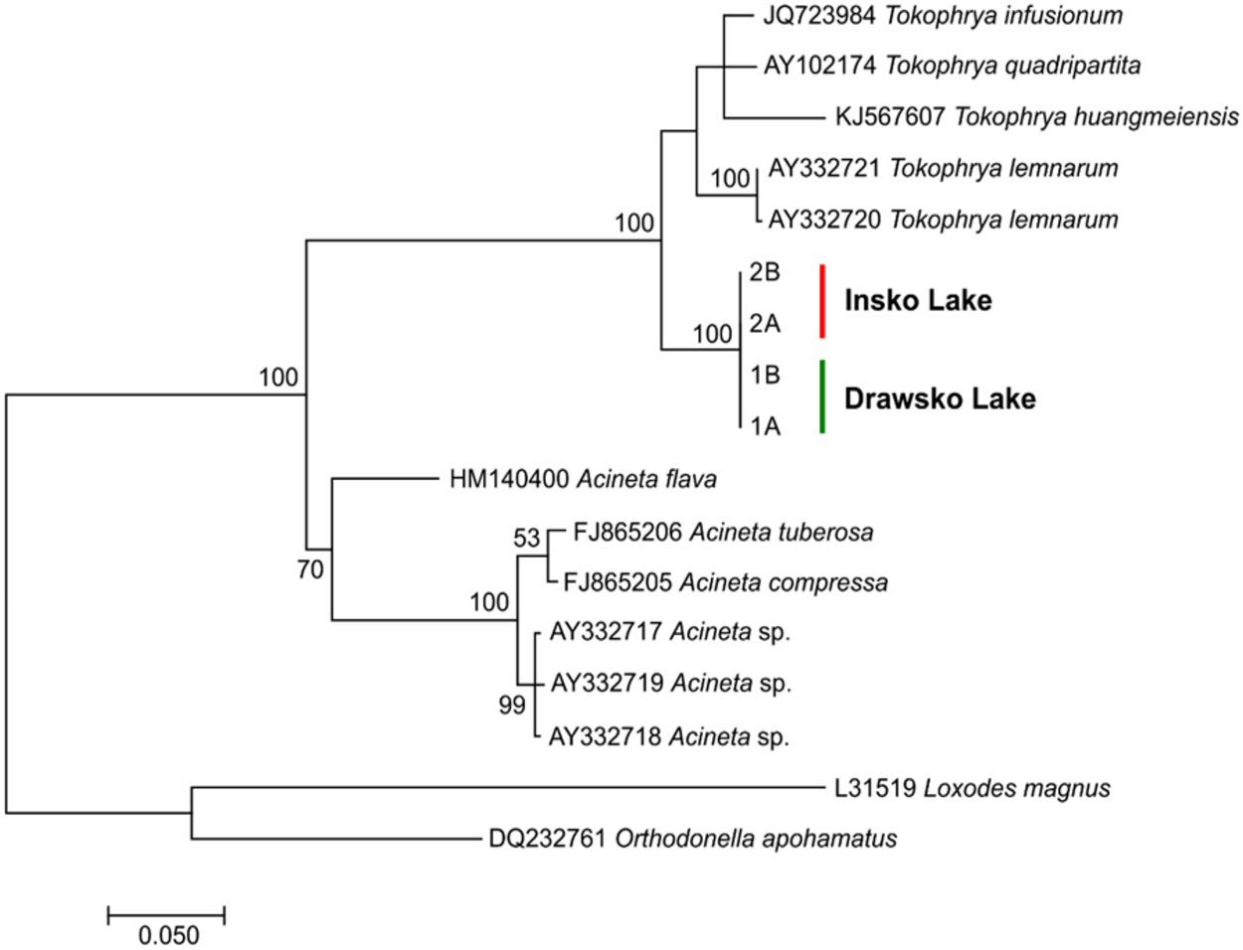
Maximum Likelihood tree based on partial 18 S rRNA sequences showing the phylogenetic relationships among the representatives of Tokophryidae and Acinetidae. Numbers at the nodes represent the bootstrap values. Only values over 50 are given.

The sequential reads determined for the collected specimens represented the same unique sequence. The final alignment was found to contain a total of 606 nucleotides (338 conservative, 249 variable, and 147 parsimony-informative), which was used for subsequent phylogenetic analyses. The topology of the ML tree also supported the monophyly of both Tokophryidae and Acinetidae with a high bootstrap value (Fig. 3). Moreover, the sequences determined in the present study formed the well-supported group in the Tokophryidae clade.

The mean value of *p*-distance calculated inside the identified clades was 0.048 ± 0.006 for Tokophryidae and 0.046 ± 0.005 for Acinetidae, while the mean value calculated for this distance between the clades was 0.175 ± 0.014. On the other hand, the values of *p*-distance determined in the pairwise comparison were found to be higher for the sequences determined in the present study (1 A, 1B, 2 A, and 2B), compared with the Acinetidae (>15%) and Tokophryidae sequences (<8%) (Table 2). As none of the records in the molecular database showed a close match to *Tokophrya* species, we confirmed that it might be a specific epibiont of *Tokophrya* genus associated with the *E. lacustris* species.

**Table 2:** Values of uncorrected *p*-distance calculated for sequence pairs. The values of standard error are given in italics. Sequences of *Loxodes magnus* and *Orthodonella apohamatus* (accession numbers: L31519 and DQ232761, respectively) were used as outgroups. The sequences that were determined in the present study are marked in bold.

	AY332720	AY332721	JQ723984	KJ567607	AY102174	**1 A, 1B, 2 A, 2B**	HM140400
AY332720		*0.002*	*0.009*	*0.010*	*0.008*	*0.010*	*0.015*
AY332721	0.002		*0.009*	*0.010*	*0.008*	*0.010*	*0.015*
JQ723984	0.047	0.045		*0.009*	*0.007*	*0.009*	*0.015*
KJ567607	0.066	0.064	0.050		*0.009*	*0.011*	*0.015*
AY102174	0.045	0.043	0.025	0.053		*0.009*	*0.015*
**1 A, 1B, 2 A, 2B**	0.066	0.064	0.057	0.078	0.055		*0.014*
HM140400	0.160	0.160	0.157	0.162	0.159	0.152	
FJ865205	0.183	0.182	0.181	0.180	0.187	0.178	0.106
FJ865206	0.173	0.171	0.170	0.173	0.180	0.171	0.102
AY332717	0.178	0.176	0.182	0.180	0.185	0.178	0.102
AY332718	0.178	0.177	0.181	0.178	0.186	0.178	0.101
AY332719	0.181	0.179	0.185	0.182	0.188	0.181	0.105
L31519	0.284	0.284	0.290	0.291	0.297	0.295	0.250
DQ232761	0.250	0.250	0.258	0.262	0.264	0.261	0.213
	FJ865205	FJ865206	AY332717	AY332718	AY332719	L31519	DQ232761
AY332720	*0.015*	*0.015*	*0.015*	*0.015*	*0.015*	*0.019*	*0.018*
AY332721	*0.015*	*0.015*	*0.015*	*0.015*	*0.015*	*0.019*	*0.018*
JQ723984	*0.015*	*0.015*	*0.015*	*0.015*	*0.015*	*0.019*	*0.018*
KJ567607	*0.015*	*0.015*	*0.015*	*0.015*	*0.015*	*0.019*	*0.018*
AY102174	*0.015*	*0.015*	*0.015*	*0.015*	*0.015*	*0.019*	*0.018*
**1 A, 1B, 2 A, 2B**	*0.015*	*0.015*	*0.015*	*0.015*	*0.015*	*0.019*	*0.019*
HM140400	*0.012*	*0.012*	*0.012*	*0.012*	*0.012*	*0.017*	*0.017*
FJ865205		*0.004*	*0.006*	*0.006*	*0.007*	*0.018*	*0.017*
FJ865206	0.011		*0.007*	*0.006*	*0.007*	*0.018*	*0.017*
AY332717	0.023	0.027		*0.002*	*0.003*	*0.018*	*0.017*
AY332718	0.022	0.025	0.004		*0.003*	*0.019*	*0.017*
AY332719	0.025	0.029	0.005	0.005		*0.019*	*0.017*
L31519	0.260	0.260	0.266	0.268	0.272		*0.016*
DQ232761	0.214	0.209	0.225	0.222	0.224	0.217	

### Infection parameters

**Figure 4:**
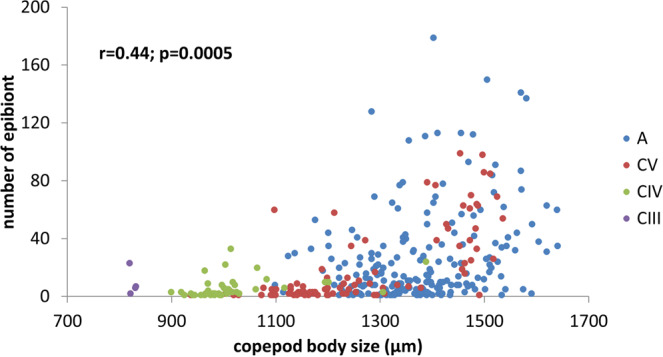
Number of *Tokophrya* specimens in relation to the body size and maturity stage of *Eurytemora*. A—adult; CIII-CV—copepodid stages.

**Figure 5:**
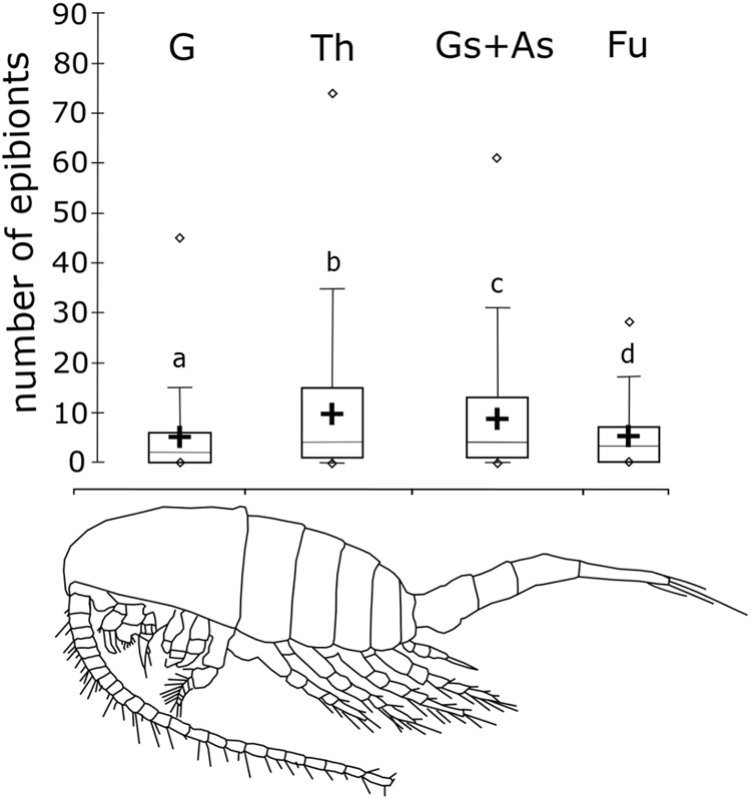
Boxplot of epibiont number among the body parts of *Eurytemora lacustris* showing preferences of *Tokophrya* to certain body parts. Different letters (a-d) indicate significant differences in infection level with *p* value <0.005 for Th and Gs+As, and *p* value <0.001 between other parts of the body. G—cephalosome; Th—pedigerous somite; Gs+As—genital somite and anal somite; and Fu—caudal ramus.

**Figure 6:**
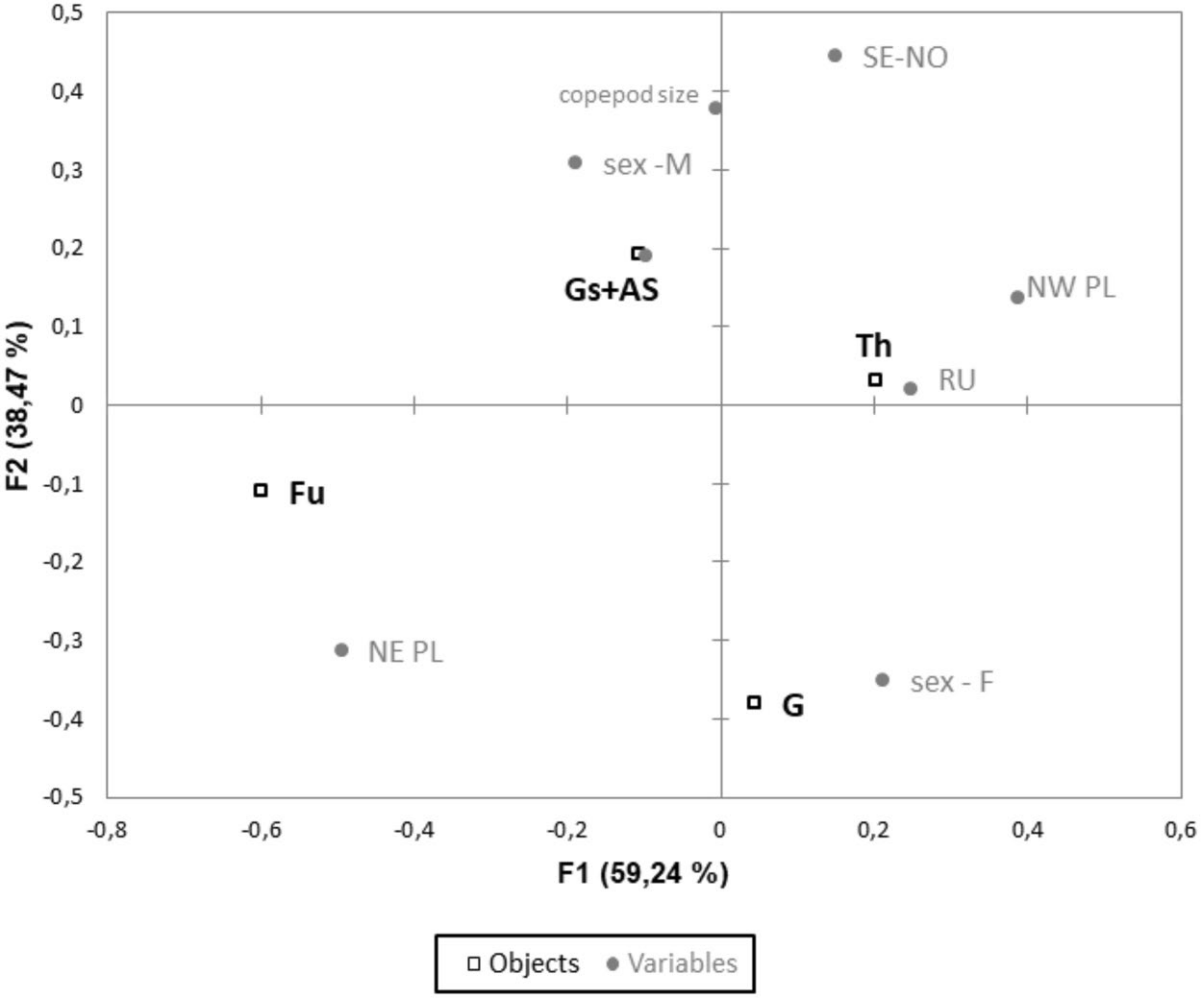
Canonical correspondence analysis ordination diagram showing the infection intensity of certain body parts of *Eurytemora* (G—cephalosome, Th—pedigerous somite, Gs+As—genital somite and anal somite, Fu—caudal ramus), with respect to sex (M—male, F—female), region (NE PL—northeastern Poland, NW PL—northwestern Poland, SE-NO—Sweden and Norway, RU—Russia), and body size of calanoids.

At all the developmental stages investigated in the present study, the copepods were found to be infected by *Tokophrya*, and the number of epibionts increased with an increase in the body size of the copepods (*r* = 0.44; *p* = 0.0005) (Fig. 4). Furthermore, adult calanoids were characterized by a statistically significantly higher level of infection than juveniles (*F*_1,349_ = 8.7; *p* < 0.0002). With respect to sex, although a higher infection rate was observed for males than females, the observed difference was not statistically significant (*F*_1,349_ = 1.2; *p* < 0.3). A Kruskal-Wallis test showed that there was a significant difference of infection level among body parts (*H*_3_ = 162.3; *p* < 0.0001). The body parts of the copepods lying near its center of gravity (Th, Gs+As) were found to be the most infected and differ from other parts of copepod body (*p* < 0.001) (Fig. 5). However, we noted that the differences in the colonization of body parts were dependent on the sex of the copepods. In males, a greater level of epibiont colonization was observed on Gs and As, whereas in females, the suctorians were the most abundant in Th (Fig. 6). The same analysis also showed strong colonization in the caudal rami of *Eurytemora* by the epibionts collected from NE Poland.

## Discussion

In the present study, a close relationship was observed between *Eurytemora lacustris* and *Tokophrya* in the entire geographical gradient of the species. It was confirmed that the suctorians occur in Polish, Lithuanian, Russian, Norwegian, and Swedish lakes, but in a previous study the epibiontic suctorians were also found in German lakes^17^. The prevalence of suctorians in the regions assessed in our study seemed to be similar and indicated differences that could be explained by seasonal fluctuations of basibionts, which was already pointed out as a key factor affecting the prevalence of epibionts on microcrustaceans^3^. The lowest prevalence noted in the Russian water bodies might have resulted due to taking into account the samples from the atypical habitat of Neva Bay which is considered to be a brackish habitat and does not probably meet the requirements for the survival of *Tokophrya*^10^. *Tokophrya* is found in lakes that meet the environmental requirements of *E. lacustris* and could be threatened by extinction in a small, isolated population. In such habitats, the decline and disappearance of the population may prevent its rebuilding. In the present study, we did not find any suctorian on *Eurytemora* in lake Cieszęcino, which is one of the smallest lakes with stable populations of *E. lacustris*. This may suggest that the interaction is likely reversible and that if significant environmental changes occur, *Tokophrya* will not be able to survive in the habitat. Moreover, rapid climate changes can potentially cause significant modulations in the preserved interaction between crustaceans and suctorians, by altering the thermal conditions of the lakes and the well-established preference of *E. lacustris* for cold waters.

Species that are relatively recently adapted to freshwater habitats, the so-called postglacial relicts, are reported to maintain their former marine adaptations that are related to life strategies and physiological and morphological characteristics. For instance, lipids and life cycle strategies described for the glacial relict *Limnocalanus macrurus* were characterized to be specific to polar species^28^. However, it seems that heritage from the former habitat may relate not only to the features of the crustacean itself but also to the accompanying species that are living in close relationship with the host, and thus, there had to be parallel changes in their adaptation to freshwater conditions. We speculate that the relationship between the copepod and the suctorian investigated in the present study dates back to the time when the ancestor of *Eurytemora* was associated with marine polar habitats. Hence, as a result of environmental changes and due to the depletion of the former habitat of *E. lacustris*, a specific epibiont was gradually adapted. Epibiontic suctorians are found to be in close interaction with marine species^11,29^, and very commonly polar species^30^. The North American lakes that inhabit the stenothermic copepods *L. macrurus* and *Epischura lacustris* are colonized by a similar or the same *Tokophrya* species^4,10^ as found in the case of *E. lacustris*. Among the species of *Eurytemora* genus, we identified suctorian epibionts only on stenothermic *E. lacustris* and did not observe suctorians on any other species of the genus. Therefore, we assume that *Tokophrya* is host-specific for stenothermic relicts including *E. lacustris*, and such preserved interactions can thus lead to the coevolution of species.

The widespread interaction of *Eurytemora* and *Tokophrya* appears to be neutral or favorable to crustacean populations, which is in contradiction to previous studies that have described that this relationship (epibiont–basibiont) is adverse to Copepoda. We suppose that the interactions between the epibionts and calanoids favor their habitat preferences to the deeper layers of the lakes. Similar to any prey, *Eurytemora* would try to avoid its predators by becoming less visible and therefore prefer to remain at relatively large depths of the lakes. At the same time, with an intensive settlement of epibionts, migration becomes energetically expensive for *Eurytemora*, and so it is optimal for them to stay in the aphotic zone of lakes. Thus, when *Eurytemora* accustomed to the lakes, its habitat preferences shifted toward metalimnion and hypolimnion. In addition, the fouling-control strategy of *Eurytemora* species facilitated the preservation of their habitat preferences. Hence, it seems that the interactions between suctorians and crustaceans may have contributed to the establishment of their preferences for cold waters that are fulfilled by the aphotic zone.

Furthermore, we found that the level of infection is related to the basibiont size, as the adults showed a higher level of infection and prevalence than the juveniles. In the adult stage, *Eurytemora* remains in the environment for the longest duration, during which their chance to be colonized is the greatest. In addition, intensive colonization of adult specimens may result from a larger body surface and possibly the release of chemicals that attract epibionts^3^. Moreover, the level of infection may depend on the frequency and length of mating. The suctorian attached to the spermatophore placed on the female genital segment (Fig. 2L) could cause infection. As copepods molt gradually (the molting process does not occur simultaneously in all parts of the body in these organisms), it seems that trans-stadial transmission also contributes to *Tokophrya* infection. Therefore, some individuals may remain on the host, allowing the epibiont population to establish on the copepods in the next stage.

Strong colonization may prevent *Eurytemora* from reaching a zone rich in food or getting out of areas that unfavorable to the copepod, such as the bottom zone where oxygen deficits may appear. This may also pose a threat to *Eurytemora*, especially when the lake becomes so strongly eutrophicated that oxygen deficits occur in the warmer periods^31^.

In the present study, CCA which was performed for analyzing the level of colonization of individual body parts by suctorians separated eastern Poland from the other assessed regions. This might be due to the highest level of infection noted in the studied lakes. In particular, the highest level of infection in NE Poland could be related to local factors, but this requires further examinations. If calanoids are strongly infected by *Tokophrya*, the middle parts of the body are colonized first, following which the infection spreads to the rest of the body^4^. Hence, the highly infected calanoids were characterized by a high settlement on all the body parts, including the caudal ramus. A previous study indicated that *T. quadripartita* colonizing the urosome or the last metasomal segment of the lightly infested *L. macrurus* but heavily infested copepods also inhabited the dorsal, ventral, and lateral surfaces of the cephalothorax^4^. The level of colonization and prevalence of *Eurytemora* were shown to be similar to those reported in the lakes of North America^4,10^. Moreover, the expected center of gravity of the copepod coincided with those parts of the body most commonly inhabited by the epibiont. In this way, the epibiont did not significantly disturb the symmetry of the copepod, posing only limited difficulties in their mobility. It should be noted that strong distal colonization could lead to severe impairment of motor functions and might even lead to the death of the host.

Our study confirmed that a specific epibiont was associated with *E. lacustris*, regardless of the region. Genetic analyses revealed that the epibiont belongs to the genus *Tokophrya*. We also found that the level of basibiont colonization is related to its size and identified that the most intensely inhabited body parts are those located near the center of the copepod body. We proposed two hypotheses regarding the relationship between the epibiont and the copepod. However, the interactions between *Eurytemora* and *Tokophrya* require further study, particularly to describe the *Eurytemora*-related epibiont species, including the *Tokophrya* individuals that are associated with the stenotherms of North America and Asia. We still do not understand why epibionts choose one particular basibiont species when there are several crustaceans, including those that are more numerous than *Eurytemora*. Assuming that epibionts had played an indirect role in shaping the life strategies of *Eurytemora*, it is interesting in our opinion to investigate whether the established relationship is and was beneficial for the survival of the species or it can be a threat to *Eurytemora* in the event of climate changes. A part of this question, including the mechanism of colonization, would be answered only by experimental research, which might help in solving the mystery of the long-term relationship between the stenothermic microcrustacean and the suctorian epibiont.
